# Human health risk ​and receptor model-oriented sources of heavy metal pollution in commonly consume vegetable and fish species of high Ganges river floodplain agro-ecological area, Bangladesh

**DOI:** 10.1016/j.heliyon.2022.e11172

**Published:** 2022-10-21

**Authors:** Tapos Kumar Chakraborty, Gopal Chandra Ghosh, Md Ripon Hossain, Md. Shahnul Islam, Ahsan Habib, Samina Zaman, Himel Bosu, Md. Simoon Nice, Monisankar Haldar, Abu Shamim Khan

**Affiliations:** a Department of Environmental Science and Technology, Jashore University of Science and Technology, Jashore 7408, Bangladesh; b Department of Computer Science and Engineering, Jashore University of Science and Technology, Jashore 7408, Bangladesh; c Environmental Laboratory, Asia Arsenic Network, Arsenic Center, Benapole Road, Krishnobati, Pulerhat, Jashore 7400, Bangladesh

**Keywords:** Metal contamination, Fish and vegetables, Human health risk, Multivariate analysis, Bangladesh

## Abstract

This study was intended to assess heavy metal contents and sources in commonly consumed vegetables and fish collected from the Jashore district of Bangladesh and to evaluate the probable human health risks via the ingesting of those vegetables and fish species. A total of 130 vegetable and fish samples were analyzed for As, Mn, Cu, Cr, Ni, and Pb concentration by an atomic absorption spectrophotometer. Metals and metalloids like As, Pb, and Cr in vegetable species were greater than the maximum allowable concentration (MAC), while Pb and cu in fish species exceeded the MAC. Pollution evaluation index values were ranges from 0.40-10.35 and 1.53–2.78 for vegetable and fish species, respectively, indicating light to serious pollution. *Lactuca sativa* followed by *Cucurbita moschata, Amaranthus gangeticus* for vegetables and *Channa punctate, Oreochromis mossambicus*, followed by *Dendrobranchiata* for fish are the most contaminated food items. The positive matrix factorization model showed that As (81.9%), Ni (48%), Cr (49.6%), Mn (46%), Pb (44.3%), and Cu (44.4%) for vegetable species and As (86.9%), Ni (90.5%), Mn (67.6%), Pb (65.3%), Cr (57%) and Cu (46.2%) for fish species were resulting from agrochemical, atmospheric emission, irrigation, contaminated feed, and mixed sources. The self-organizing map and principle component analysis indicates three spatial patterns e.g., As–Mn–Cu, Pb–Cr, and Ni in vegetables and As–Mn–Cr, Cu–Ni, and Pb in fish samples. The THQ values for single elements were less than 1 (except As for vegetables and Pb for fish species) for all food items but the HI values for all of the vegetables (2.18E+00 to 2.04E+01) and fish (1.07E+00 to 9.39E+00) samples were exceeded the USEPA acceptable risk level (HI > 1E+00). While the cancer risks only induced by As for all vegetables and fish species, which exceeded the USEPA safe level (TCR>1E-04). Sensitivity analysis indicates that metal concentration was the most responsible factor for carcinogenic risk.

## 1 Introduction

Food safety is considered a greatest significant issue by scientists due to public health concerns. Mainly, the human body is beings exposed to heavy metals via food consumption and about 90% of exposure are occurred through eating than other routes such as dermal and inhalation (Mansour et al., 2009). At present, heavy metals associated with health risk by consumption of contaminated foodstuffs have been drawing more attention among researchers (Shaheen et al., 2016). Toxic metals and metalloids are among the most severe hazardous components in the environment and have universal concerns due to their severe poisonous properties, high staying power, and potential toxic character (Chakraborty et al., 2021; Rahman et al., 2018). Conversely, toxic metals and metalloids have helpful and harmful effects on living organisms. It can be categorized as vital (Co, Zn, Cu, Mo,Fe, Mn, Se etc.) and non-vital elements (As, Cd Pb, Hg, Ni, Cr, etc.) (Ghosh et al., 2020; Shaheen et al., 2016). The vital metals and metalloids could be toxic if ingested in excess amounts while non-vital metals and metalloids are highly harmful even at low dose for a long time exposure and express diverse health effects such as carcinogenic, non-carcinogenic, mutagenic, teratogenic, etc. Chromium and Ni are identified to create a range of respiratory disorderness, such as lung infection, fibrosis, bronchial asthma, tumors, and epithelial cell injury (Forti et al., 2011; Ghosh et al., 2018), while the elevated level of Cu can create primary biliary cirrhosis, chronic hepatitis, liver, and kidney damage (Fuentealba and Aburto, 2003). Lead altered the pathological state in organs and the cerebrospinal nervous system, trigger to damage to skeletal, circulatory, and enzymatic, and reduce children intelligence quotients (IQ). Poor reproductive capacity, cancer, bone injury, high blood pressure, and respiratory and kidney disease are began by cadmium poisoning (Rahman and Islam, 2009; Proshad et al., 2018), whereas inorganic As can create cancer, and is the available noxious form of As (Ali et al., 2020; Chakraborty et al., 2022). Heavy metal contaminations in aquaculture and farmland have been getting global consideration, particularly in the least developed countries like Bangladesh. The sources of metals and metalloids in the agricultural fields and aquatic ecosystem, and even in the common food items (vegetable and fish species) of Bangladesh are connected with diverse manmade causes including fast industrialization, effluent irrigation, usage of bio-solid, excess use of metals and metalloids-contained fertilizers, toxic metals and metalloids contaminated fish feeds and improper management of foodstuff (during store and transportation) (Sarker et al., 2017; Zakir et al., 2021). As an aquatic organisms fish is being highly exposed to toxic metals and metalloids, which is not only danger to fish species but also to human health by consumption of contaminated fish (Islam et al., 2015a) because in Bangladesh about 60% of total animal protein demand make up by fish as well as it is a good source of essential nutrients (vitamins, minerals and fatty acids) (DoF, 2019; Islam et al., 2016). Conversely, vegetables have played a key role in human nutrition. Fresh vegetables contain a lot of micro and macro nutrients for the upkeep of improved health as well as the prevention and managing of numerous diseases. Vegetables encompass both vital and non-vital metals over a great variety of concentrations during manufacturing, harvesting, transportation, and selling (Haque et al., 2021), which is enough to create severe health hazards to both animals and humans due to consuming these metals contaminated vegetables (Sankar et al., 2006) Fish and vegetables are the two key food items, which regularly consumed by the Bangladeshi people. Numerous studies (Aysha et al., 2017; Haque et al., 2018; Zakir et al., 2020) found a high concentration of diverse toxic metals in those foodstuffs. Various diseases may perhaps develop due to the consumption of metals and metalloids contaminated food items by the reduction in immunological defenses, reduced psychological behavior, malnutrition-related illness, and a high occurrence of upper digestive tract (Zakir et al., 2020). Metals and metalloids cannot be degraded or destroyed; they can alter their chemical forms (Martí-Cid et al., 2008). Consequently, at present, metals and metalloids addition to the human body from these commonly consumes vegetable and fish species is a complex issue. Thus, heavy metal contamination in commonly consume food items are a critical concern for food quality and safety assurance in Bangladesh. So, the risk assessment of toxic metals and the benefit of commonly consumed vegetables and fish via daily dietary ingestion is a very vital issue: the presence of the main source of vital elements and nutrients (Martí-Cid et al., 2008). Different techniques have been used for human health risks (eg. carcinogenic and non-carcinogenic) and foodstuff contamination level assessment (Ghosh et al., 2020; Islam et al., 2018). At the present time, various researchers applied numerous methods for contaminated foodstuff-induced human health risk to decrease unreliability or even over and under assessment. For this purpose, Monte Carlo simulations are a quantifiable risk assessment approach to measure the possibility of the spread of risk (Chakraborty et al., 2022). However, detailed source distribution of metals and metalloids in foodstuff is far away from scientific attention although which is important for human health concerns. Plotting pollution zones by using a single analytical method are challenging due to diverse and complex pollution sources (Bhuiyan et al., 2021; Kuerban et al., 2020). To find out the precise and reliable results of heavy metal sources, this study applied a Self-organizing map (SOM), receptor model (e.g. PMF) and Principle component analysis (PCA). In this study, the advantage of assimilating these source-oriented models might be a new method for numerous pollution sources distributed in the foodstuffs of Bangladesh. In Bangladesh, several authors try to find out the metals and metalloids contain in the different foodstuffs (Zwolak et al., 2019; Islam et al., 2018; Sultana et al., 2017; Shaheen et al., 2016) but this is the first study that considered the metals and metalloids in commonly consume fish and vegetable species together with their probable sources and potential human health risks using receptor models and multivariate approaches, respectively. Consequently, this study investigates (i) the concentration of metals and metalloids (As, Pb, Cu, Cr, Ni, and Mn) in commonly consume foodstuff (vegetable and fish species), (ii) evaluates the probable health risks allied with metals and metalloids through ingestion of these food items and (iii) to measure the possible sources of metals and metalloids in these vegetable and fish species. This study was mainly focused on Jashore district, situated in the western part of the Ganges river floodplain agro-ecological zone. Due to favorable environmental conditions, diverse vegetables and fish are produced throughout the year in Jashore. After meeting local demand, these foodstuffs are supplied to other regions of the country (Nath, 2013). At present, excess agrochemicals and commercial fish feed are applied to produce more vegetables and fish for getting more profit, beside this area is close to different point and non-point sources.

## 2 Materials and methods

### 2.1 Fish and vegetable sample collection and preparation

**Figure 1:**
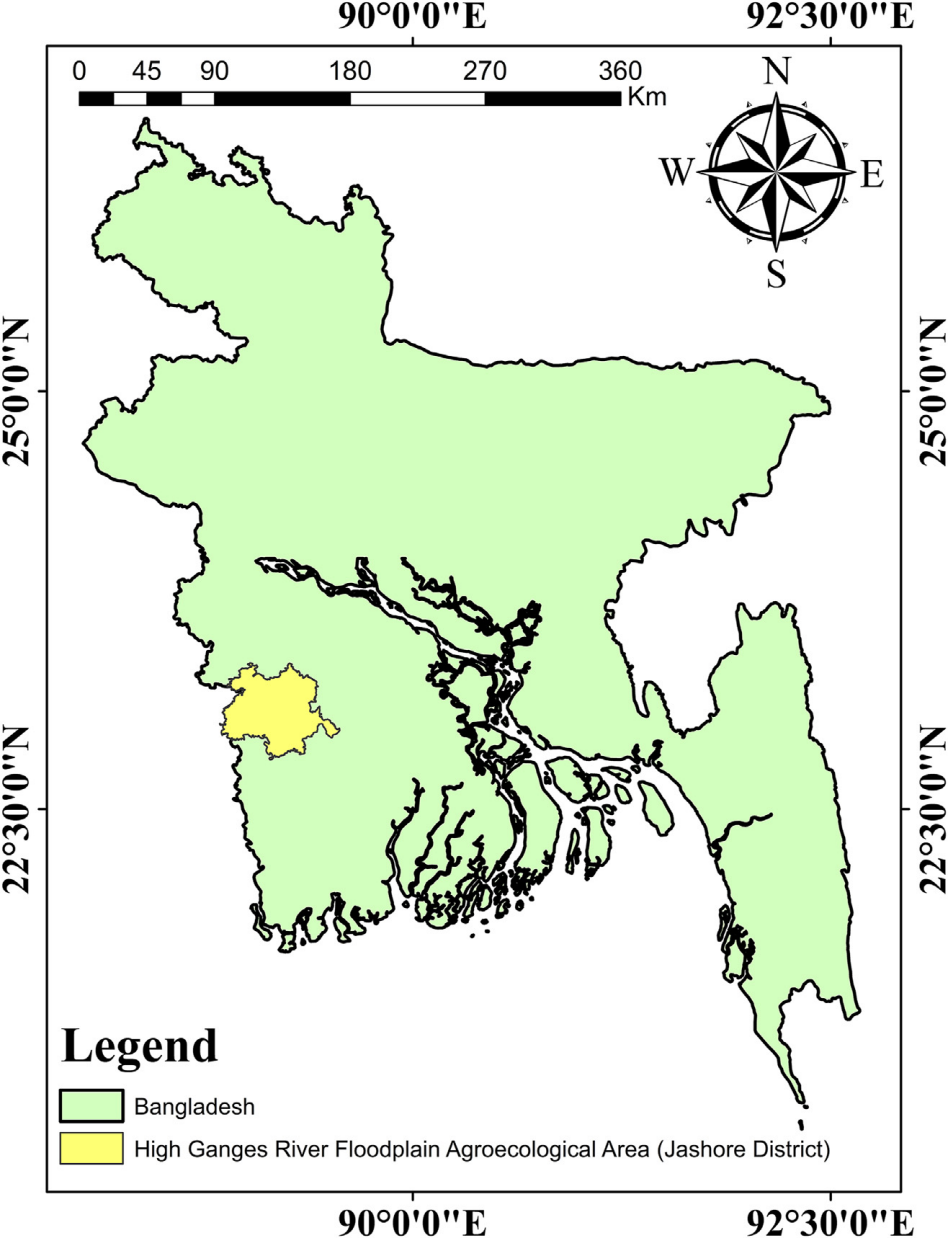
Map of the study area.

In the present study, fish and vegetable, samples were collected from through the Jashore district (Figure 1), situated in the western part of the high Ganges river floodplain agro-ecological area, Bangladesh during the period of September–October 2021. In total, 75 samples of fifteen different vegetable species, *Momordica charantia* (Bitter gourd), *Cucumis sativus* (Cucumber), *Solanum melongena* (Brinjal), *Solanum lycopersicum* (Tomato), *Spinacia oleracea* (Spinach), *Daucus carota subsp. Sativus* (Carrot), *Cucurbita moschata* (Pumpkin), *Musa paradisiaca. linn* (Banana), *Trichosanthes dioica* (Pointed gourd), *Lactuca sativa* (Red Amaranth), *Amaranthus gangeticus* (green chili), *Lagenaria siceraria* (Bottle gourd), *Solanum tuberosum* (Potato), *Carica papaya* (Papaya), and *Amaranthus gangeticus* (Amaranths leaves), as well as 55 samples of eleven fish species, *Oreochromis mossambicus* (Indian Tilapia), *Dendrobranchiata* (Prawns), *Puntius chola* (Swamp barb), *Trichogaster chuna* (Honey gourami), *Channa punctata* (Spotted snakehead), *Anabas cobojius* (Climbing gourami), *Heteropneustes fossilis* (Stinging cat fish), *Cirrhinus cirrhosis* (Mrigal), *Labeo rohita* (Indian Rui/Rohu), *Labeo bata* (Bata), and *Pangasius pangasius* (Yellowtail catfish) were collected from the different place of Jashore district, Bangladesh. Sample selection and the listing were completed according to the key food approach and choice of the inhabitants in the study area.

Separate polythene/zipper bags with detailed indications were used during sample collection. At the end of sample collection, all samples were taken to the laboratory, where, vegetable samples were prudently washed by deionized water, and the consumable parts of vegetables were cut into tiny sizes, and then dried in an oven-at 80 °C temperature, to achieve constant weight. The samples were ground by a crushing machine after appropriate drying. For fish samples, at first cleaning with deionized water, then parted into flesh (consumable) and bone (non-consumable). Fish flesh once more parted into muscle and abdomen, and then muscle parts were oven dried at 80 °C temperature awaiting the samples achieved constant weight. After completing drying, all samples (vegetable and fish species) were crushed and ground individually by a mechanical crusher and kept in a sealed clean container with detailed indications in freezing state until further analysis. The precaution was taken during the grinding process to avoid any contamination. International, national, and institutional standard procedures were applied through the whole study for handing and use of fish species. This research does not encompass any issues concerning human participants done by any of the authors. These studies evaluate the heavy metals concentration in food items and calculate the human health risk based on the assessed concentrations. No further toxicological experiments were conducted on any other living species.

### 2.2 Preparation of extract for heavy metal analyses

Vegetable and fish samples were digested by following the wet oxidation method utilizing a tri-acid mixture [HNO_3_ (69%):H_2_SO_4_ (98%): HClO_4_ (70%) = 5:1:1] as explained by Allen et al. (1986). For digestion, accurately 1.00 g of crushed sample was taken into a 250 mL conical flask and digested with 15 mL of the tri-acid mixture at 180–200 °C temperature until a clear solution was achieved. During sample digestion conical flask was covered by a watch glass for controlling the possible loss of volatile elements and after certain time intervals conical flask glass wall was washed with 0.1 M HNO_3_ solution to reduce the possible adsorption trace metals on glass. Then, the digested solution was cooled to about 25 °C temperature, then filtered by Whatman 42 paper (pre-washed with 0.1 M HNO_3_) and diluted to 50 mL with double-distilled water, and blank samples were also ready by using a similar procedure. Laboratory grade chemicals (Merck, Germany) and double-distilled water were utilized throughout all the experiments.

### 2.3 Determination of heavy metals concentration in samples and analytical quality assurance

The concentration of As, Cr, Pb, Ni, Cu, and Mn in the samples were investigated using a hydride initiator, graphite furnace, and air-acetylene flame Atomic-absorption-spectrophotometer (AAS) (Model: AA-7000, SHIMADZU, Japan), prepared with particular element hollow-cathode lamp as a light source at the wavelength of 193.70, 357.90, 283.3, 232.00, 324.80 and 279.5 nm, respectively. The detection limit for As was 0.0003 mg/kg, while for Cu, Cr, Mn, Pb, and Ni was 0.013–0.070 mg/kg. Standard solution (1000 mg/L; Sigma Aldrich, Switzerland) was used for instrumental calibration. The analysis results were stated as mg/kg for vegetable and fish samples. Double distilled water was used through the all experiment. Clean and dried equipment’s and glassware’s were used for experimental work. Certified reference material (CRM) DORM-4 Fish protein, used as a CRM for heavy metals. The CRM was collected from the National Research Council (NRC), Canada. The recoveries were within 88–110% (Table S1) of the certified values showing a good relationship between the certified and detected values.

### 2.4 Pollution load evaluation method

The average pollution load index (APLI) approach is used to evaluate the possibly harmful effects of studied metals in exposed vegetable and fish species (Liu et al., 2021), the following formula is used in Eq. (1).

(1) $$APLI=\frac{1}{n}\;\sum_{n=1}^{n}\frac{C_{i}}{S_{i}}$$

where APLI indicate metals and metalloids contamination in fish and vegetable samples; n represent the number of metals and metalloids; C_i_ is the mean value of metals and metalloids measured in fish and vegetable samples; S_i_ is the maximum admissible concentration (MAC) of metals and metalloids (Table 2); the greater the PI value suggested that the fish and vegetable is not consumable (Liu et al., 2021). Based on the metals and metalloids concentration in fish and vegetable species, the PI < 0.1, 0.1–0.2, 0.2–0.5, 0.5–0.7, 0.7–1.0, >1.0, unpolluted; micro pollution; lightly polluted moderately, polluted; moderately polluted; heavily pollution; seriously polluted, respectively.

### 2.5 Estimation of human health risk

#### 2.5.1 Assessment of daily metal intakes (EDMI)

The EDMI (mg kg/bw/day) was considered to measure metal induced health risk via ingestion of vegetable and fish species. The calculation is done by following Eq. (2)

(2) $$EDMI=\frac{FIR\times C}{BW}$$

Where FIR represent food intake rate (g/person/day), C denotes metal concentrations in fish and vegetable species (mg/kg, fw), and BW means the mean body weight (60 kg for adults) (BBS, 2015). For adult inhabitants, 170.04 g vegetable and 59.91 g fish was considered as the daily food ingestion rate (HIES, 2017).

#### 2.5.2 Non-carcinogenic risk

In this study, USEPA proposed a health risk assessment method was applied to assess the non-carcinogenic and carcinogenic health risk of the local peoples (USEPA, 1989). The non-carcinogenic health hazards for every single metal via fish and vegetable ingestion were evaluated by the target hazard quotient (THQ). On the other hand, the total target hazard quotient (TTHQ) was applied to measure the combined effect of all metals and metalloids, and calculation was done by using the following equation (3) and equation (4), respectively.

(3) $$THQ=\frac{C\times FIR\times EF\times ED}{BW\times AT\times RfD}\times 10^{-03}$$

(4) $$TTHQ={THQ}_{metal\;1}+{THQ}_{metal\;2}+{THQ}_{metal\;3}+\ldots \ldots \ldots \ldots \ldots \ldots \ldots ..+{THQ}_{metal\;n}$$

Where EF and ED indicate the exposure frequency (365 days/year) and exposure duration (70 years), respectively of human lifetime (USEPA, 1991); BW indicate body weight and AT is the averaging time for non-carcinogens (AT = 365×ED). The R*f*D is oral reference dose. The oral reference dose for As, Mn, Cr, Ni, Cu and Pb, was 0.0003, 0.14, 1.5, 0.02, 0.04 and 0.004 mg/kg/day, respectively (USEPA, 2010). If the THQ <1 or THQ >1, show no noticeable, and major non-carcinogenic health hazards of a single element for the inhabitants, respectively. Additionally, *TTHQ* < 1 designates no remarkable adverse non-carcinogenic health risk, and *TTHQ* > 1 shows major adverse non-carcinogenic health risk.

#### 2.5.3 Carcinogenic risk

Target cancer risk (TCR) was evaluated as the slow possibility of an individual creating cancer over a lifespan by exposing cancer-creating element (USEPA, 2010). Eq. (5) was applied for assessing the TCR:

(5) $$TCR=\frac{C\times FIR\times EF\times ED\times CSF}{BW\times AT}\times 10^{-03}$$

CSF carcinogenic slope factor of As and Pb was 1.5 and 8.5 9E-03 mg/kg/day, respectively (USEPA, 2010). CR are characterized into five categories; <1E-06, >1E-06 to <1E-05, >1E-05 to <1E-04, >1E-04 to <1E-03, and >1E-03, suggested very low, low, medium, high and very high, respectively (USEPA, 1999).

### 2.6 Self-organizing map (SOM) analysis

SOM, a type of artificial neural network established by Kohonen (1982), was effectively utilized in multiple data analysis including grouping, estimating, and forecasting with unverified training (Nakagawa et al., 2020). In this study, SOM was applied for arrangement identification of metals and metalloids in fish and vegetable species. The component planes deal with the graphical inspection of associations between variables. The analogous gradients characterize a positive association, while the anti-parallel offers a negative association in the element planes. However, there are no recommended rules for selecting the neuron quantities in the output layer. SOM analysis done using the following Eq. (6)

(6) $$m=5\times \sqrt{n\;}$$

Where m denotes the amount of SOM map nodes, and n is the quantity of input data. Math lab R2017 was used for SOM analysis.

### 2.7 Positive matrix factorization (PMF)

PMF (EPA PMF version 5.0) is a mathematical source oriented model (USEPA, 2014), used to distribute the source of metals and metalloids in fish and vegetables. Mathematically it can be stated as Eq. (7):

(7) $$X_{iy}=\sum_{j=1}^{a}g_{ia}f_{ay\;}+e_{iy}$$

Where, x_iy_ is the i^th^ species value calculated in the y^th^ sample; f_ay_ is the input of a^th^ source to y^th^ sample; g_ia_ is the value of i^th^ species from y^th^ source, and e_iy_ is the methodical error. The objective of this model was to determine the values for g_ia_ and f_ay_, which greatest repeat the estimated value x_iy_. These data were perfected up to the last Q value was attained, where Q is well-defined as Eq. (8).

(8) $$Q\left(A\right)=\sum_{y=1}^{m}\sum_{i=1}^{n}\left(\frac{e_{iy}}{\alpha_{iy}}\right)$$

Here the α_iy_ states the “uncertainty” in the i^th^ species of sample number y. The data under the minimum determination limit (MDL) are replaced with the approaches using Eqs. (9) and (10).

(9) $$\alpha_{iy}=\frac{5}{6}\times MDL\;\left(X_{iy\;}\leq MDL\right)$$

(10) $$\alpha_{iy}=\left(0.05\times X_{iy}\right)+MDL\;\left(X_{iy\;}\geq MDL\right)$$

In this model data was run 20 times randomly along with diverse number of factors (varied from 3 to 6) until desired result obtained (i. e. lowest Q values, greater R^2^ values and perfect definable factors) (Bhuiyan et al., 2021). Q (robust) and Q (True) were discussed appropriately for very runs, and run 13 was designated for factor taking out where steps were started at least 127.

### 2.8 Monte Carlo Simulation

The probable carcinogenic risk assessment was achieved by applying a Monte Carlo Simulation for cancer-causingc metals ingested through consumption of vegetable and fish species. This is the most familiar approaches utilize to detect the variability and uncertainties of risk-based calculation (USEPA, 1997). Every simulation was passed by 10,000 random trails of each input data for confirming the reliability of the simulated results. In this present study, the cancer risks for As and Pb (average, 5th, and 95th percentiles) was taken from the TCR probability distribution. Moreover, a sensitivity analysis was applied to evaluate which input variable influence the risk calculation. Crystal Ball software (v. 11.1.2.4) developed by Oracle Co. was used for the assessment of possibility risk and sensitivity analysis.

### 2.9 Statistical analysis

In this study SPSS V.16.0 (SPSS, USA) used for statistical analysis. The average and standard deviations of the toxic metals and metalloids contents in food items samples were calculated. The multivariate statistical tools; principal component analysis (PCA) Pearson correlation matrix (PCM) and cluster analysis (CA) were applied to find the complete facts of the dataset and the dispersal of metals and metalloids in the samples according to their similarities or dissimilarities and concentration. For PCA, the average value of every variable was utilize, where eigenvalue and loading value > 1 and >0.5, respectively for every principal element was taken from the analysis table for the description of the analysis results.

## 3 Results and discussion

### 3.1 Concentrations of metals in vegetables

The concentrations of metals and metalloids [mg/kg dw (dry weight)] in 15 vegetable species are presented in Table 1. In total, the mean concentrations of metals and metalloids in 15 vegetables were in the descending order of Mn > Cu > Ni > Cr > Pb > As. Arsenic (As) is omnipresent in the environment by manmade and natural sources. Elevated level of As expose might be create diverse health hazards including dermatological, hematologic and neurologic disorders, etc (Tchounwou et al., 2012). The average concentrations of As in vegetable species varied from 0.67 mg/kg Red Amaranth (*Lactuca sativa*) to 0.08 mg/kg Banana (*Musa paradisiaca. linn*) (Table 1). The average concentration of As in the vegetables was greater than the FAO/WHO guideline (0.1 mg/kg) except for Amaranths leaves (*Amaranthus gangeticus)* and Banana (*Musa paradisiaca. linn*), representing As might pose risk through ingestion of these contaminated vegetables. The study from Bangladesh by Islam et al. (2016) stated that As in vegetables varied from 0.13 to 0.52 mg/kg. The range of As concentrations in vegetables from different sampling point of Dhaka and Faridpur area Bangladesh was 0.01–0.2 mg/kg 40]. Manganese (Mn) is essential for the living being because it influence various enzymatic reactions (Bhat and Gómez-López 2014), but high level of Mn intake are responsible for permanent neurological disorder tremors, difficulty walking, and facial muscle spasms (Yoon et al., 2007). The highest and lowest concentration of Mn was found in 12.69 mg/kg Papaya (*Carica papaya)* and 68.77 mg/kg Red Amaranth (*Lactuca sativa*), respectively. Shaheen et al. (2016) found the range of Mn in vegetable species from the *Bangladesh agro*-ecological zone was 6.98 mg/kg to 28.35 mg/kg. The study from Jashore, Bangladesh by Ara et al. (2018) indicated that Mn in vegetables ranged from 11.33 to 130.31 mg/kg. High concentration of chromium (Cr) is able to create respiratory tract irritation, pulmonary sensitization or even lung, nasal, and sinus cancer (Hasan et al., 2021). The mean concentration of Cr in the studied vegetable samples was 1.53 mg/kg and ranges from 0.85-3.50 mg/kg, where the probable source of Cr in those vegetables was the application of excess agrochemicals in the crop fields. In previous literature, Shaheen et al. (2016) showed that the concentrations range of Cr from the *Bangladeshi agro*-ecological zone was 1.11–0.29 mg/kg. The concentration of nickel (Ni) varied from 0.98 Spinach (*Spinacia oleracea*) to 4.29 mg/kg Red leaf lettuce (*Lactuca sativa*) (Table 1).

**Table 1:** Metals and metalloids concentrations (mg/kg dw) ranges (mean ± SD) in vegetables in the study area, (n = 75).

English name	Scientific name	As	Mn	Cu	Ni	Pb	Cr
Bitter gourd	*Momordica charantia*	0.32 ± 0.16	15.42 ± 6.54	12.22 ± 4.67	1.38 ± 0.56	0.09 ± 0.06	0.99 ± .056
		0.11–0.51	9.01–24.12	7.11–18.22	0.87–2.22	0.04–0.20	0.05–1.54
Cucumber	*Cucumis sativus*	0.24 ± 0.25	23.21 ± 6.20	17.32 ± 3.73	1.46 ± 1.17	0.13 ± 0.16	1.35 ± 0.91
		0.03–0.67	16.80–31.23	12.10–21.65	0.03–3.01	0.02–0.40	0.23–2.61
Tomato	*Solanum lycopersicum*	0.33 ± 0.38	23.45 ± 8.27	12.20 ± 4.21	2.04 ± 0.98	0.11 ± 0.08	1.02 ± 0.62
		0.05–0.98	12.21–31.20	5.54–16.28	0.56–3.21	0.02–0.21	0.23–1.89
Brinjal	*Solanum melongena*	0.18 ± 0.15	19.74 ± 7.86	16.41 ± 8.29	2.01 ± 0.91	0.18 ± 0.24	0.85 ± 0.31
		0.02–0.40	11.43–31.29	9.22–26.54	0.67–3.23	0.01–0.60	0.56–1.25
Spinach	*Spinacia oleracea*	0.37 ± 0.42	53.77 ± 24.33	17.39 ± 7.49	0.98 ± 0.40	0.17 ± 0.17	2.10 ± 0.99
		0.09–1.11	22.91–80.45	10.78–29.09	0.45–1.49	0.02–0.44	0.56–3.21
Carrot	*Daucus carota subsp. sativus*	0.15 ± 0.12	16.46 ± 4.74	16.51 ± 6.59	1.55 ± 0.74	0.13 ± 0.16	0.98 ± 1.20
		0.01–0.31	10.33–21.65	8.45–23.56	0.87–2.56	0.01–0.40	0.23–3.11
Pumpkin	*Cucurbita moschata*	0.13 ± 0.11	18.23 ± 6.03	14.76 ± 9.65	4.20 ± 1.78	4.91 ± 3.13	1.42 ± 0.93
		0.02–0.31	11.23–26.41	9.19–31.87	2.10–6.33	1.45–9.02	0.45–2.67
Banana	*Musa paradisiaca.linn*	0.08 ± 0.09	18.14 ± 9.77	11.82 ± 5.35	1.65 ± 1.17	0.03 ± 0.04	1.11 ± 0.60
		0.01–0.23	9.12–31.23	8.12–21.09	0.08–3.22	0.00–0.09	0.65–2.10
Pointed gourd	*Trichosanthes dioica*	0.18 ± 0.10	16.77 ± 8.44	9.88 ± 6.27	1.97 ± 1.00	0.14 ± 0.15	1.43 ± 1.18
		0.08–0.33	5.56–28.77	2.33–19.11	0.87–3.33	0.01–0.33	0.08–3.01
Red Amaranth	*Lactuca sativa*	0.67 ± 0.31	68.77 ± 31.38	23.96 ± 7.92	4.29 ± 2.92	0.62 ± 0.44	1.63 ± 1.64
		0.23–1.01	23.45–103.20	10.76–30.87	0.67–8.11	0.10–1.02	0.06–4.01
green chili	*Amaranthus gangeticus*	0.16 ± 0.22	20.55 ± 10.55	12.37 ± 7.10	1.27 ± 1.03	0.12 ± 0.08	1.52 ± 0.74
		0.01–0.51	3.21–31.49	5.09–23.88	0.33–2.88	0.02–0.23	0.91–2.65
Bottle gourd	*Lagenaria siceraria*	0.14 ± 0.13	15.40 ± 11.24	9.64 ± 7.44	1.99 ± 1.53	0.20 ± 0.11	0.99 ± 0.51
		0.01–0.33	2.11–31.02	2.11–21.09	0.23–4.19	0.07–0.33	0.34–1.77
Potato	*Solanum tuberosum*	0.13 ± 0.14	17.01 ± 10.29	9.57 ± 4.50	1.30 ± 0.86	0.12 ± 0.10	1.92 ± 1.06
		0.01–0.32	2.09–29.06	2.66–15.09	0.33–2.38	0.03–0.28	0.73–3.01
Papaya	*Carica papaya*	0.12 ± 0.08	12.69 ± 8.59	9.99 ± 5.53	2.65 ± 3.68	0.13 ± 0.10	2.20 ± 1.75
		0.01–0.23	3.18–25.09	4.23–18.09	0.12–9.09	0.02–0.30	0.34–4.09
Amaranths leaves	*Amaranthus gangeticus*	0.09 ± 0.12	51.37 ± 28.40	9.82 ± 11.13	1.02 ± 1.13	4.67 ± 3.31	3.50 ± 1.95
		0.01–0.30	21.63–90.56	1.89–28.76	0.12–2.87	0.56–8.99	1.22–5.92
Maximum allowable conc. (MAC) (FAO/WHO, 2011)		0.1	-	40	10	0.1	2.3

**Table 2:** Metals and metalloids concentrations (mg/kg dw) ranges (mean ± SD) in fish species in the study area, (n = 55).

English name	Scientific name	As	Mn	Cu	Ni	Pb	Cr
Indian Tilapia	*Oreochromis mossambicus*	2.44 ± 1.61	26.56 ± 10.79	6.07 ± 4.29	0.03 ± 0.06	3.72 ± 6.86	0.26 ± 0.51
		0.45–4.24	10.11–37.11	1.77–12.09	0.00–0.07	1.78–6.86	0.01–0.67
Prawns	*Dendrobranchiata*	0.51 ± 0.35	4.25 ± 2.13	8.62 ± 4.88	0.55 ± 0.56	4.57 ± 2.17	0.14 ± 0.12
		0.09–1.01	1.91–6.99	2.01–14.89	0.00–1.29	1.23–6.55	0.03–0.33
Swamp barb	*Puntius chola*	0.03 ± 0.03	2.53 ± 1.12	4.71 ± 4.31	0.02 ± 0.03	3.32 ± 1.49	0.18 ± 0.27
		0.00–0.08	1.01–4.33	1.77–12.09	0.00–0.07	1.78–4.89	0.01–0.67
Honey gourami	*Trichogaster chuna*	0.03 ± 0.03	9.79 ± 3.59	3.26 ± 1.97	0.02 ± 0.02	4.75 ± 2.60	0.19 ± 0.27
		0.00–0.08	3.98–4.33	1.04–12.09	0.00–0.07	1.04–4.89	0.01–0.67
Spotted snakehead	*Channa punctata*	0.04 ± 0.04	13.23 ± 6.74	5.57 ± 3.96	0.01 ± 0.02	6.21 ± 3.17	0.20 ± 0.13
		0.00–0.09	5.59–20.87	1.01–10.22	0.00–0.04	1.09–9.02	0.07–0.40
Climbing gourami	*Anabas cobojius*	0.05 ± 0.09	12.07 ± 8.84	4.45 ± 4.32	0.02 ± 0.03	5.16 ± 3.45	0.10 ± 0.03
		0.00–0.20	2.01–21.09	0.23–11.11	0.00–0.07	1.09–9.11	0.04–0.12
Stinging cat fish	*Heteropneustes fossilis*	0.08 ± 0.08	6.85 ± 4.34	3.54 ± 2.24	0.02 ± 0.04	5.08 ± 3.03	0.09 ± 0.07
		0.00–0.20	2.01–12.09	1.38–7.11	0.00–0.1.	1.09–8.13	0.00–0.20
Mrigal	*Cirrhinus cirrhosus*	0.44 ± 0.31	4.66 ± 3.77	5.36 ± 4.04	0.03 ± 0.04	4.59 ± 2.59	0.10 ± 0.10
		0.11–0.90	1.05–10.11	1.09–10.23	0.00–0.10	1.76–8.56	0.00–0.20
Indian Rui/Rohu	*Labeo rohita*	0.08 ± 0.08	7.89 ± 6.02	4.45 ± 2.88	0.04 ± 0.04	4.38 ± 2.77	0.13 ± 0.07
		0.00–0.20	1.23–17.29	1.38–7.71	0.00–0.10	1.11–8.45	0.03–0.20
Bata	*Labeo bata*	0.24 ± 0.33	5.42 ± 3.59	4.81 ± 3.99	0.06 ± 0.08	4.55 ± 1.90	0.15 ± 0.13
		0.00–0.80	1.31–10.87	0.12–10.50	0.00–0.20	2.01–6.74	0.00–0.30
Yellowtail catfish	*Pangasius pangasius*	0.34 ± 0.29	4.35 ± 3.50	0.91 ± 0.43	0.10 ± 0.17	3.42 ± 2.03	0.15 ± 0.26
		0.01–0.80	1.23–10.01	0.23–1.38	0.00–0.40	1.06–6.26	0.00–0.60
Maximum Allowable Conc. (MAC) (FAO/WHO, 2002)		1.0	1.0	4.5	0.8	0.5	1.0

Nickel is an essential micronutrient and help to human metabolism, but expose at high level may causes headache, cough, cardiac and kidney illnesses, lung fibrosis, lung and nasal cancer (Hasan et al., 2021). The mean concentration of Ni in the studied vegetable samples was 1.98 mg/kg, lower than the FAO/WHO guideline (10 mg/kg), which showed that the studied vegetables were free from Ni contamination. The maximum mean concentration of copper (Cu) was detected in Red leaf lettuce (*Lactuca sativa*) (29.96 mg/kg) followed by Potato (*Solanum tuberosum*) (9.57 mg/kg) (Table 1). In a recent study in the Jashore district, Bangladesh, Ara et al. (2018) found Cu concentrations between 1.12-30.80 mg/kg in different vegetable species. The highest mean concentration of lead (Pb) was assessed in Pumpkin (*Cucurbita moschata*) (4.91 mg/kg) and the lowest in Banana (0.03 mg/kg) (Table 1). The mean concentration of Pb in all studied vegetables was 0.78 mg/kg, higher than the FAO/WHO permissible limit (0.1 mg/kg), designated that vegetables were contaminated by Pb. Haque et al. (2021) found an elevated concentration of Pb (ranges from 0.81-3.93 mg/kg) in vegetables from different site in Dhaka and Faridpur region, Bangladesh. The concentration of metals and metalloids in vegetable samples were varied might be due to the variation of metals and metalloids absorption or accumulation capacities, diverse growth periods and influencing of soil properties like pH, organic matter, cation exchange capacity, and relations of soil–plant root–microbes (Gebrekidan et al., 2013). Additionally, uses of untreated wastewater as irrigation, deposition of metals and metalloids contain atmospheric fallout from diverse burning place, using of chemical during crops storage and handling and excess uses of agrochemicals and fertilizers (Bhuiyan et al., 2011; Rahman et al., 2013).

### 3.2 Metal concentrations in fish species

In this study, fish muscles were considered for the valuation of heavy metals content because this is the most preferable edible portion among Bangladeshi people. The average concentrations of As, Pb, Cr, Ni, Cu, and Mn in eleven diverse fish species are presented in Table 2. In total, the average concentrations of heavy metals in fish species displayed the downward order of Mn > Cu > Pb > As > Cr > Ni. The elevated level of As was noticed in Indian Tilapia (*Oreochromis mossambicus*) (2.44 mg/kg) followed by Prawns (*Dendrobranchiata*) (0.51 mg/kg). The average concentration of all fish species was 0.39 mg/kg, lower than the FAO/WHO guideline (1.0 mg/kg). From Patuakhai district, Bangladesh, Islam et al. (2015a) found 0.04–0.8 mg/kg concentration of As in fish species. Mn was assessed in eleven species where the maximum concentration (26.56 mg/kg) was found in *Indian Tilapia (Oreochromis mossambicus)*, and the lowermost concentration (2.53 mg/kg) was detected in *Swamp barb (Puntius chola)*. This study found an elevated level of Mn than previous studies (1.69–2.99 mg/kg) (Hasan et al., 2021). Copper (Cu) is a vital nutrient for human body; however, great Cu expose can lead to diverse health difficulties such as diarrhea, headaches, bloodlessness, liver, kidney injury, Wilson's disease and abdominal irritation (Bhat and Gómez-López 2014). The content of Cu in the analyzed samples varied from 0.91 mg/kg Yellowtail catfish (*Pangasius pangasius*) to 8.62 mg/kg Prawns (Dendrobranchiata) (Table 2). The average value of Cu in all fish samples was 4.71 mg/kg, which exceeded the FAO/WHO standard guideline (4.5 mg/kg), indicating that fish were contaminated by Cu. This study found a greater quantity of Cu in fish species as compare with other studies, which conducted on St. Martin island (0.3–2.23 mg/kg) and Paira River (0.10–2.2 mg/kg) (Islam et al., 2015a; Baki et al., 2018). The average concentration of Ni was 0.08 mg/kg, where the uppermost value was found in Prawns (Dendrobranchiata) (0.55 mg/kg). The concentration of Ni in all studied fish species were within the limit of FAO/WHO standard guideline (0.8 mg/kg). Islam et al. (2015b) found 0.04–1.4 mg/kg Ni in the fish species of Bogra district, Bangladesh. Among the studied fish species, the *Spotted snakehead (Channa punctata)* revealed the highest mean concentration (6.21 mg/kg), whereas *Swamp barb (Puntius chola) showed* the lowest mean concentration (3.32 mg/kg). The average value (4.52 mg/kg) of all fish species was 9.04 times higher than the FAO/WHO recommended safe value (0.5 mg/kg). Approximate 2.76–4.63 mg/kg concentration of Pb was found in the market available fishes of Noakhali, Bangladesh (Ahmed et al., 2019). The elevated level of Pb exposure can lead to creating several health hazards such as digestive and nephritic illness, fiber bundle, and histopathological disorderness (Mansour, 2014). In this study, diverse concentration of metals and metalloids were observed in the fish species due to the difference of fish age, size, growth rate, feeding behaviors, excess uses of commercial feeds and influencing by local pollution sources (Dural et al., 2007; Al Sayegh et al., 2012).

### 3.3 Estimated daily intake (EDI)

A dietary exposure method is a dependable tool for evaluating a population's diet based on their nutrient intake rate, bioactive compounds, and contaminants (WHO, 1985). This study evaluates the dietary exposure of metals and metalloids via the ingesting of vegetables and fish item in the daily diet of the adult people. The EDIs of metals and metalloids (As, Ni, Cu, As, and Pb) were assessed based on the mean concentration of every element in every food and the particular ingesting rate (Santos et al., 2004). The EDI of the considered metals and metalloids from the ingestion of fish and vegetables are presented in Tables 3 and 4. In fish and vegetable species, total values of EDI exhibited the downward order of Mn > Cu > Pb > As > Cr > Ni and Mn > Cu > Ni > Cr > Pb > As, respectively. The total EDI value of the metals and metalloids via ingestion of these vegetables (Table 3) were greater than the EDI value from fish ingestion (Table 4), suggesting that vegetables and fish are seriously contaminated by metals and metalloids in the area of the Jashore district in Bangladesh. The vegetable and fish samples were lesser than the MTDI, according's to Shaheen et al. (2016), Haque et al. (2021) and JECFA (2003).

**Table 3:** Estimated dietary intake (EDI) (mg/kg-bw/day; ww) of metals and metalloids due to consumption of vegetables.

Vegetable species	Estimated Daily Intakes (EDIs) (mg/kg-bw/day; ww)					
	As	Mn	Cu	Ni	Pb	Cr
*Momordica charantia*	9.01E-04	4.37E-02	3.46E-02	3.92E-03	2.66E-04	2.79E-03
*Cucumis sativus*	6.89E-04	6.58E-02	4.91E-02	4.14E-03	3.63E-04	3.83E-03
*Solanum lycopersicum*	9.32E-04	6.65E-02	3.46E-02	5.79E-03	3.06E-04	2.89E-03
*Solanum melongena*	5.04E-04	5.59E-02	4.65E-02	5.70E-03	4.99E-04	2.40E-03
*Spinacia oleracea*	1.04E-03	1.52E-01	4.93E-02	2.78E-03	4.70E-04	5.96E-03
*Daucus carota subsp. sativus*	4.14E-04	4.66E-02	4.68E-02	4.39E-03	3.57E-04	2.78E-03
*Cucurbita moschata*	3.60E-04	5.17E-02	4.18E-02	1.19E-02	1.39E-02	4.04E-03
*Musa paradisiaca.linn*	2.15E-04	5.14E-02	3.35E-02	4.66E-03	9.07E-05	3.14E-03
*Trichosanthes dioica*	5.21E-04	4.75E-02	2.80E-02	5.58E-03	4.08E-04	4.06E-03
*Lactuca sativa*	1.89E-03	1.95E-01	6.79E-02	1.22E-02	1.77E-03	4.61E-03
*Amaranthus gangeticus*	4.65E-04	5.82E-02	3.51E-02	3.61E-03	3.46E-04	4.30E-03
*Lagenaria siceraria*	4.11E-04	4.36E-02	2.73E-02	5.63E-03	5.72E-04	2.82E-03
*Solanum tuberosum*	3.77E-04	4.82E-02	2.71E-02	3.70E-03	3.40E-04	5.43E-03
*Carica papaya*	3.43E-04	3.60E-02	2.83E-02	7.52E-03	3.74E-04	6.23E-03
*Amaranthus gangeticus*	2.61E-04	1.46E-01	2.78E-02	2.88E-03	1.32E-02	9.93E-03
Total	9.32E-03	1.11E+00	5.78E-01	8.44E-02	3.33E-02	6.52E-02
Maximum tolerable daily intake (MTDI)	1.30E-01	2.00E+00	3.00E+01	3.00E-01	2.10E-01	2.00E-01

**Table 4:** Estimated dietary intake (EDI) (mg/day) of metals and metalloids due to consumption of fish.

Fish species	Estimated Daily Intakes (EDIs) (mg/kg-bw/day; ww)					
	As	Mn	Cu	Ni	Pb	Cr
*Oreochromis mossambicus*	2.43E-03	2.65E-02	6.06E-03	2.80E-05	3.72E-03	2.64E-04
*Dendrobranchiata*	5.05E-04	4.25E-03	8.60E-03	5.51E-04	4.57E-03	1.42E-04
*Puntius chola*	3.20E-05	2.53E-03	4.70E-03	1.80E-05	3.31E-03	1.84E-04
*Trichogaster chuna*	2.80E-05	9.77E-03	3.26E-03	1.80E-05	4.74E-03	1.88E-04
*Channa punctata*	4.39E-05	1.32E-02	5.57E-03	1.40E-05	6.20E-03	1.96E-04
*Anabas cobojius*	4.59E-05	1.20E-02	4.44E-03	2.00E-05	5.15E-03	9.79E-05
*Heteropneustes fossilis*	7.59E-05	6.84E-03	3.54E-03	2.40E-05	5.07E-03	9.39E-05
*Cirrhinus cirrhosus*	4.35E-04	4.65E-03	5.35E-03	3.39E-05	4.59E-03	9.99E-05
*Labeo rohita*	7.79E-05	7.88E-03	4.44E-03	3.79E-05	4.37E-03	1.26E-04
*Labeo bata*	2.44E-04	5.41E-03	4.80E-03	5.99E-05	4.55E-03	1.46E-04
*Pangasius pangasius*	3.43E-04	4.34E-03	9.13E-04	9.99E-05	3.41E-03	1.48E-04
*Total*	4.27E-03	9.75E-02	5.17E-02	9.05E-04	4.97E-02	1.68E-03
Maximum tolerable daily intake (MTDI)	1.00E+00	-	4.50E+00	9.00E-01	3.00E-01	1.00E+00

### 3.4 Non-carcinogenic and carcinogenic risk

The non-carcinogenic (HQ) risk of As, Cr, Mn, Ni, Cu, and Pb with carcinogenic risk (cancer risk) of As, and Pb for consumption of metals and metalloids contaminated vegetables and fishes are presented in Tables 5 and 6, respectively. The THQ value of As was higher than recommended value (THQ>1) for almost all of the vegetable species without two species (*Musa paradisiaca. linn and Amaranthus gangeticus)*, showing As might pose a major non-carcinogenic health risk to humans, where highest As THQ was found in *Lactuca sativa* (6.29E+00). Besides this, other metals, viz*.,* Mn (2.57E-01 to 1.09E+00), Cu (7.08E-01 to 1.23E+00), Ni (3.76E-01 to 1.39E-01), Pb (2.27E-02 to 3.48E+00) and Cr (4.15E-03 to 3.08E-04), occupied the below or above the acceptable limits (THQ>1). The descending order of non-carcinogenic risk of analyzed metals was As > Cu > Pb > Mn > Cr > Ni. According to HI value of vegetable species ranges from 2.21E+00 to 1.04E+01, indicating all vegetable species exceeded the safe level (HI > 1) (Table 5). Islam et al. (2016) and Haque et al. (2021) found that As was the main contributing metal for non-carcinogenic health risk in the Bogra, Dhaka and Faridpur region, Bangladesh, respectively. The descending ranking order of HI for vegetable species was *Lactuca sativa > Cucurbita moschata > Amaranthus gangeticus > Spinacia oleracea > Solanum lycopersicum > Momordica charantia > Cucumis sativus > Solanum melongena > Daucus carota subsp. Sativus > Trichosanthes dioica > Amaranthus gangeticus > Lagenaria siceraria > Carica papaya > Solanum tuberosum > Musa. paradisiaca. linn* (Table 5). The THQ value of Pb was higher than the accepted level (THQ>1) for most of the fish species without three species (*Puntius chola, Pangasius pangasius, and Oreochromis mossambicus)*, Pb could contribute to the severe issue for residents of the study area. The highest Pb THQ was found in *Channa punctata* (1.55E+00). On the other hand, other metals, viz*.,* As (9.32E-02 to 8.11 + 00) Mn (6.987E-02 to 1.89E-01), Cu (8.15E-02 to 1.52–01), Ni (8.99E-04 to 1.40E-03), and Cr (1.22E-04 to 1.30E-04) occupied the below or above the acceptable limits (THQ>1) (Table 6). The descending order of non-carcinogenic risk of analyzed metals in was As > Pb > Cu > Mn > Ni > Cr. According to HI value of vegetable species ranges from 1.07E+00 to 9.39E+00, representing all of the fish species exceeded the safe level (HI > 1). The descending ranking order of HI for fish species were *Oreochromis mossambicus > Dendrobranchiata > Cirrhinus cirrhosis > Labeo bata > Pangasius pangasius > Channa punctate > Heteropneustes fossilis > Anabas cobojius > Labeo rohita > Trichogaster chuna > Puntius chola* (Table 6). According to the non-carcinogenic risk assessment result As, Pb, and Cu are the most common contaminants in the studied vegetable and fish species. Additionally, this analysis also indicated that continuous consumption of these vegetables and fish species may lead to non-carcinogenic health risks in the future. *Lactuca sativa* and *Oreochromis mossambicus* showed the highest HI among all of the vegetable and fish species, respectively, so too much and regular ingestion of these species is not safe for the residents of the study area. In this study, As and Pb were considered for target carcinogenic risk (TCR) calculation, meanwhile these metals may stimulate both non-carcinogenic and carcinogenic health risks based on the exposure level. USEPA classified As and Pb as carcinogen groups A and B2, respectively for animal studies. The TCR of As and Pb for adults through consumption of studied vegetable and fish species are presented in Tables 7 and 8, respectively. In vegetables, TCR values for As ranged from 3.22E-04 to 2.83E-03, indicating all of the vegetable species have As-associated cancer risk. TCR values for Pb varied from 7.71E-07 to 1.18E-04, where except for two species (*Musa paradisiaca. linn* and *Lactuca sativa*) other vegetable species were free from Pb-related cancer risk (Table 8). In fish, TCR values for As were between 1.19E-05 to 3.65E-03, showing that without four fish species (*Puntius chola, Trichogaster chuna, Channa punctate, Anabas cobojius*) other rest of the species have As associated cancer risk (Table 8).

**Table 5:** Target hazard quotient (THQ) of metals and metalloids due to consumption of vegetables.

Vegetable species	THQ						
	As	Mn	Cu	Ni	Pb	Cr	HI(TTHQ)
*Momordica charantia*	3.00E+00	3.12E-01	8.66E-01	1.96E-01	6.66E-02	1.86E-03	4.44E+00
*Cucumis sativus*	2.30E+00	4.70E-01	1.23E+00	2.07E-01	9.07E-02	2.55E-03	4.29E+00
*Solanum lycopersicum*	3.11E+00	4.75E-01	8.64E-01	2.90E-01	7.65E-02	1.92E-03	4.82E+00
*Solanum melongena*	1.68E+00	4.00E-01	1.16E+00	2.85E-01	1.25E-01	1.60E-03	3.65E+00
*Spinacia oleracea*	3.48E+00	1.09E+00	1.23E+00	1.39E-01	1.18E-01	3.98E-03	6.06E+00
*Daucus carota subsp. sativus*	1.38E+00	3.33E-01	1.17E+00	2.19E-01	8.93E-02	1.86E-03	3.19E+00
*Cucurbita moschata*	1.20E+00	3.69E-01	1.05E+00	5.95E-01	3.48E+00	2.69E-03	6.69E+00
*Musa paradisiaca.linn*	7.15E-01	3.67E-01	8.38E-01	2.33E-01	2.27E-02	2.09E-03	2.18E+00
*Trichosanthes dioica*	1.74E+00	3.40E-01	7.00E-01	2.79E-01	1.02E-01	2.71E-03	3.16E+00
*Lactuca sativa*	6.29E+00	1.39E+00	1.70E+00	6.08E-01	4.42E-01	3.08E-03	1.04E+01
*Amaranthus gangeticus*	1.55E+00	4.16E-01	8.76E-01	1.81E-01	8.64E-02	2.86E-03	3.11E+00
*Lagenaria siceraria*	1.37E+00	3.12E-01	6.83E-01	2.82E-01	1.43E-01	1.88E-03	2.79E+00
*Solanum tuberosum*	1.26E+00	3.44E-01	6.78E-01	1.85E-01	8.50E-02	3.62E-03	2.55E+00
*Carica papaya*	1.14E+00	2.57E-01	7.08E-01	3.76E-01	9.35E-02	4.15E-03	2.58E+00
*Amaranthus gangeticus*	8.71E-01	1.04E+00	6.96E-01	1.44E-01	3.31E+00	6.62E-03	6.07E+00
**Total**	3.11E+01	7.91E+00	1.44E+01	4.22E+00	8.33E+00	4.35E-02	6.60E+01

**Table 6:** Target hazard quotient (THQ) of metals and metalloids due to consumption of fish.

Fish species	THQ						
	As	Mn	Cu	Ni	Pb	Cr	Hl (TTHQ)
*Oreochromis mossambicus*	8.11E+00	1.89E-01	1.52E-01	1.40E-03	9.29E-01	1.76E-04	9.39E+00
*Dendrobranchiata*	1.68E+00	3.03E-02	2.15E-01	2.76E-02	1.14E+00	9.45E-05	3.10E+00
*Puntius chola*	1.07E-01	1.81E-02	1.17E-01	8.99E-04	8.28E-01	1.22E-04	1.07E+00
*Trichogaster chuna*	9.32E-02	6.98E-02	8.15E-02	8.99E-04	1.19E+00	1.25E-04	1.43E+00
*Channa punctata*	1.46E-01	9.44E-02	1.39E-01	6.99E-04	1.55E+00	1.30E-04	1.93E+00
*Anabas cobojius*	1.53E-01	8.61E-02	1.11E-01	9.99E-04	1.29E+00	6.52E-05	1.64E+00
*Heteropneustes fossilis*	2.53E-01	4.89E-02	8.85E-02	1.20E-03	1.27E+00	6.26E-05	1.66E+00
*Cirrhinus cirrhosus*	1.45E+00	3.32E-02	1.34E-01	1.70E-03	1.15E+00	6.66E-05	2.77E+00
*Labeo rohita*	2.60E-01	5.63E-02	1.11E-01	1.90E-03	1.09E+00	8.39E-05	1.52E+00
*Labeo bata*	8.12E-01	3.87E-02	1.20E-01	3.00E-03	1.14E+00	9.72E-05	2.11E+00
*Pangasius pangasius*	1.14E+00	3.10E-02	2.28E-02	4.99E-03	8.53E-01	9.85E-05	2.06E+00
**Total**	1.42E+01	6.96E-01	1.29E+00	4.52E-02	1.24E+01	1.12E-03	2.87E+01

**Table 7:** Target carcinogenic risk (TCR) of metals and metalloids due to consumption of vegetables.

Vegetable species	TCR		
	As	Pb	TTCR
*Momordica charantia*	1.35E-03	2.26E-06	1.35E-03
*Cucumis sativus*	1.03E-03	3.08E-06	1.03E-03
*Solanum lycopersicum*	1.40E-03	2.60E-06	1.40E-03
*Solanum melongena*	7.56E-04	4.24E-06	7.60E-04
*Spinacia oleracea*	1.56E-03	4.00E-06	1.56E-03
*Daucus carota subsp. sativus*	6.21E-04	3.04E-06	6.24E-04
*Cucurbita moschata*	5.39E-04	1.18E-04	6.57E-04
*Musa paradisiaca.linn*	3.22E-04	7.71E-07	3.23E-04
*Trichosanthes dioica*	7.81E-04	3.47E-06	7.84E-04
*Lactuca sativa*	2.83E-03	1.50E-05	2.85E-03
*Amaranthus gangeticus*	6.97E-04	2.94E-06	7.00E-04
*Lagenaria siceraria*	6.16E-04	4.87E-06	6.21E-04
*Solanum tuberosum*	5.65E-04	2.89E-06	5.68E-04
*Carica papaya*	5.15E-04	3.18E-06	5.18E-04
*Amaranthus gangeticus*	3.92E-04	1.13E-04	5.05E-04
**Total**	1.40E-02	2.83E-04	1.43E-02

**Table 8:** Target carcinogenic risk (TCR) of metals and metalloids due to consumption of fishes.

Fish species	TCR		
	As	Pb	TTCR
*Oreochromis mossambicus*	3.65E-03	3.16E-05	3.68E-03
*Dendrobranchiata*	7.58E-04	3.88E-05	7.97E-04
*Puntius chola*	4.79E-05	2.81E-05	7.60E-05
*Trichogaster chuna*	4.19E-05	4.03E-05	8.22E-05
*Channa punctata*	6.59E-05	5.27E-05	1.19E-04
*Anabas cobojius*	6.89E-05	4.38E-05	1.13E-04
*Heteropneustes fossilis*	1.14E-04	4.31E-05	1.57E-04
*Cirrhinus cirrhosus*	6.53E-04	3.90E-05	6.92E-04
*Labeo rohita*	1.17E-04	3.72E-05	1.54E-04
*Labeo bata*	3.65E-04	3.86E-05	4.04E-04
*Pangasius pangasius*	5.15E-04	2.90E-05	5.44E-04
**Total**	6.40E-03	4.22E-04	6.82E-03

On the other hand, the TCR values for Pb varied from 2.81E-05 to 5.27E-05, suggesting that all the fish species are free from Pb-related cancer risk. In the case of total target carcinogenic risk (TTCR) for vegetable and fish species were 3.32E-04 to 2.85E-03 and 7.60E-05 to3.68E-03, respectively (Tables 7 and 8), representing that the populations have lifetime cancer risk by consuming these vegetable and fish species.

Based on the total target cancer risk (TTCR) the descending order of vegetable and fish species were *Lactuca sativa* > *Spinacia oleracea* > *Solanum lycopersicum* > *Momordica charantia* > *Cucumis sativus* > *Trichosanthes dioica* > *Solanum melongena* > *Amaranthus gangeticus* > *Cucurbita moschata* > *Daucus carota* subsp. Sativus > *Lagenaria siceraria* > *Solanum tuberosum* > *Carica papaya* > *Amaranthus gangeticus* > *Musa paradisiaca*. linn and *Oreochromis mossambicus* > Dendrobranchiata > *Cirrhinus* cirrhosis > *Pangasius pangasius* > *Labeo bata* > *Heteropneustes fossilis* > *Labeo rohita* > *Channa* punctate > *Anabas cobojius* > *Trichogaster chuna* > *Puntius chola*, respectively.

According to the TTCR assessment result, the possible health risk for the study area population due to metal exposure via ingestion of fish and vegetables should not be overlooked. In this study, other food sources are not considered for the health risk evaluation. Similar outcome also found from other studies (Jia et al., 2018; Yousafzai et al., 2010; Zakir et al., 2021).

### 3.5 Pollution evaluation indices

**Figure 2:**
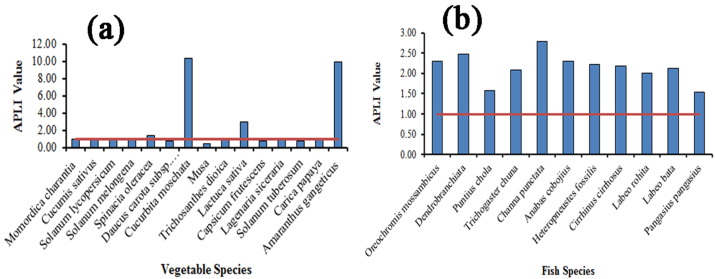
APLI value in vegetable (a) and fish (b) species in the study area.

This study applied APLI to measure the amount of the contamination level in vegetable and fish samples. In analyzed samples APLI values were ranges from 0.40-10.35 for vegetables and 1.53–2.78 for fish species (Figure 2a), indicating light to serious and serious pollution for vegetable and fish species, respectively. For vegetable samples highest APLI value found in *Cucurbita moschata* (10.35), and *Amaranthus gangeticus* (9.91) followed by *Lactuca sativa* (2.93) shows seriously polluted by studied heavy metals, while all of the fish samples in serious pollution category and maximum PI values found in *Channa punctate* (2.78), *Dendrobranchiata* (2.48) and *Oreochromis mossambicus* (2.31) for fish species, indicates serious pollution by studied heavy metals (Figure 2b). Generally, diverse feeding habits stored elevated level of heavy metals in the fishes (Yousafzai et al. 2010; Jia et al. 2018). Hasan et al. (2021) found a high contamination level in foodstuffs in their study.

### 3.6 Probabilistic health risk and sensitivity assessment

**Figure 3:**
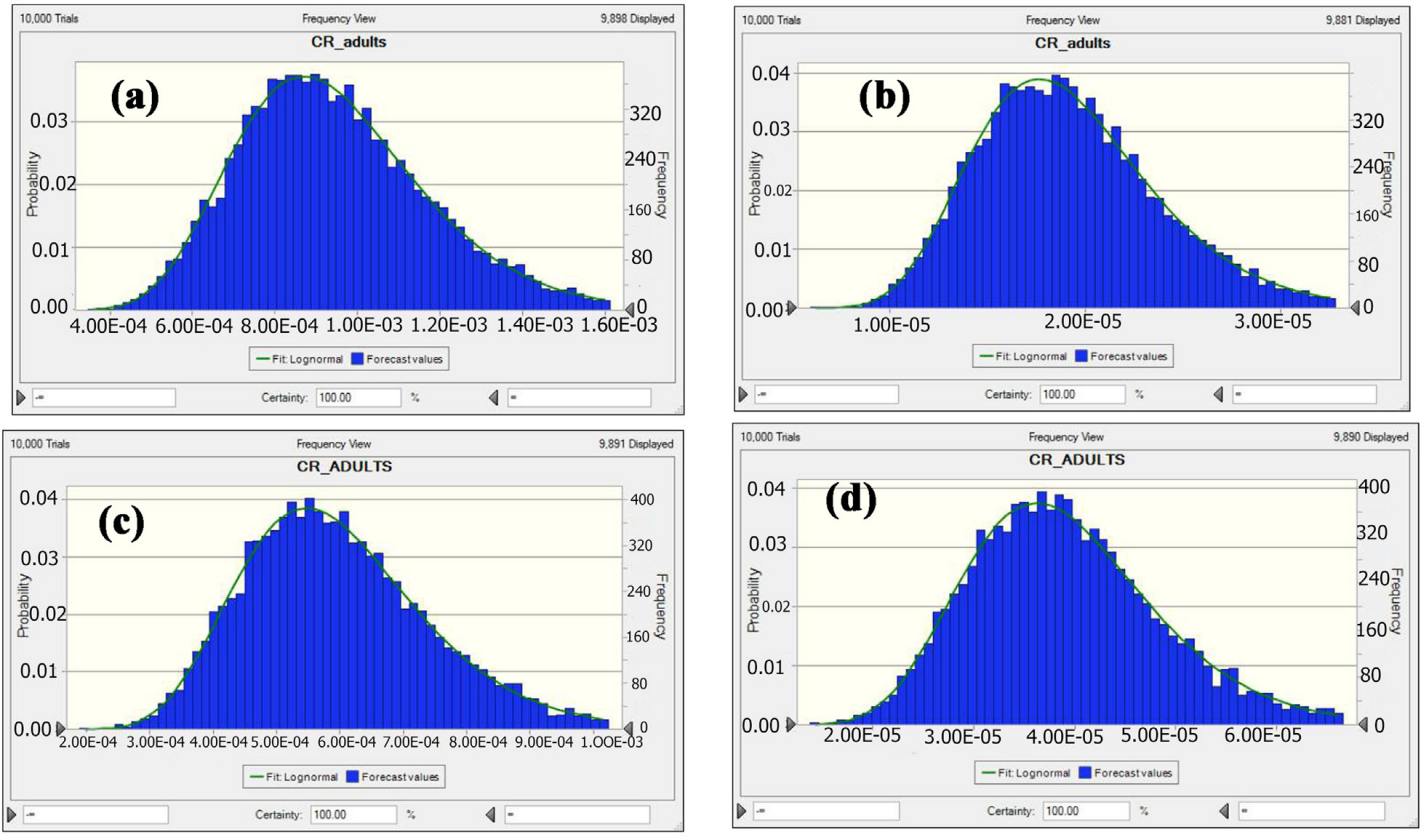
Predicted probability distribution results of the target carcinogenic risk (TR) for vegetable [(a) As and (b) Pb] and fish [(c) As and (d) Pb].

This study found that the average probability of TCR for As and Pb were 9.47E-04 ± 2.37E-04 and 1.93E-05 ± 4.82E-06 for vegetables (Figure 3a, b), and 5.95E-04 ± 1.49E-04 and 3.91E-05 ± 9.81E-06 for fish species, respectively (Figure 3c, d).

The 5th and 95th percentile values of vegetable species were detected at 6.06E-04 and 1.38E-03 for As and 1.24E-05 and 2.80E-05 for Pb. While the 5th and 95th percentile values of fish species were found at 3.82E-04 and 8.76E-04 for As and 2.52E-05 and 5.68E-05 for Pb. Based on the USEPA (USEPA, 1991) standard, the mean, 5th, and 95th percentile values of As surpassed the accepted values (>1E-04), indicating that about 95% of people would be exposed to high cancer risk from vegetables and fishes consumption. Conversely, Pb shows the accepted level of cancer risk from the mean, 5th, and 95th percentile values of Pb for TCR. Furthermore, As can be considered the significance heavy metals due to its carcinogenic effects. The significance of the input variables related to the TCR calculation was evaluated by sensitivity analysis.

**Figure 4:**
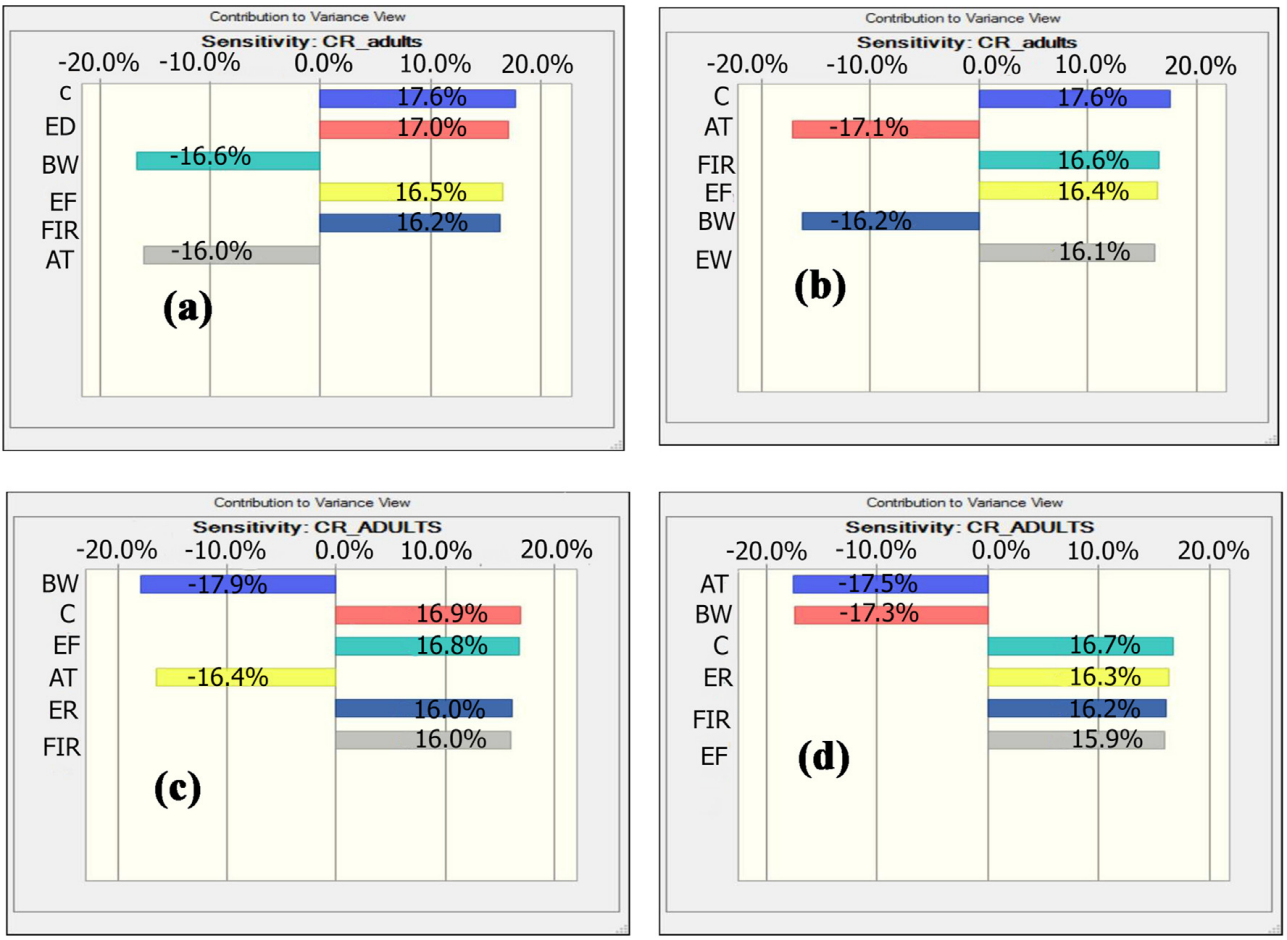
Sensitivity analysis of the target carcinogenic risks for vegetable [(a) As and (b) Pb] and fish [(c) As and (d) Pb].

The results shown that As and Pb concentration is the utmost vital factors in the TCR values for vegetable and fish species (Figure 4a and b). For vegetables, As and Pb induced TCR calculation, showed C, ED, EF, and FIR positive effects with 17.6, 17.0, 16.5, and 16.2%; 17.6, 16.1, 16.4, and 16.6%, respectively. Conversely, BW and AT indicate negative effects with a percentage of -16.6 and -16.0 for As and -16.2 and -17.1% for Pb, respectively. On the other hand, for fish C (16.9%), EF (16.8%), ED (16.0%) and FIR (16.0%) show positive effect for As as well as Pb induced TCR calculation represent positive effect for C (16.7%), EF (15.9%), ED (16.3%) and FIR (16.2%), while BW and AT revealed negative effect for As and Pb: -17.9%, -16.4% and -17.5%, -17.3%, respectively (Figure 4c, d). Finally, this study designates that metal concentration is highly liable for cancer risk assessment. Haque et al. (2021) found that concentration is the main influencing factor for cancer risk among the exposures.

### 3.7 Multivariate analysis

Multivariate statistical analysis tools help in observing and analyzing complex data. In this study, PCA, PCM, and CA are applied to find out the diverse origin of heavy metals in foodstuffs and their association. The values of Kaiser-Meyer-Olkin and Bartlett were 0.444, approx. Chi-Square = 47.828 (df = 15, P = 0.000) and 0.393, approx. Chi-Square = 27.53 (df = 15, P = 0.025) for vegetable and fish species, respectively (Figure S1). PCA analysis of metals and metalloids in the vegetables and fish species illuminates the cumulative variance of the first three axes 91.10 % (vegetables) and 88.53 % (fish). For vegetables, the first principal component (PC1) contributed 43.03% of the total variance and loading with As, Cu, and Mn (Figure S1a), which shows mutual association in the cluster diagram (Figure S1c) with a significant positive correlation among them (As–Mn = 0.684, As–Cu = 0.744) [Table S3]. The PC2 component accounted for 30.12% of the total variance and showed positive loading for Cr and Pb, additionally, CA and PCM indicate a similar association between Cr and Pb. The third group comprised of 17.96% variance of Ni for vegetables, which were mainly contributed by lithogenic sources (Iqbal and Shah, 2011). For fishes, PC1 contributes 41.47% of the total variance for As, Cr, and Mn, which are found in the same cluster in CA and PCM shows a strong positive association between As–Mn = 0.767 and As–Cr = 0.616 (Figure S1 and Table S3). Ni and Cu are present in PC 2 with 27.13% variance, where CA and PCM (Ni–Cu = 0.594) support these outcomes. The PC3 component accounted for 19.90% of the total variance and showed positive loading for Pb, which is individually located in the PCM, CA (Figure S1b, d and Table S3) and shows no correlation among other metals (Table S3) suggesting that the Pb had a distinct geochemical or anthropogenic origin (e.g. fish feed, sewage water, etc.). The multivariate analysis result showed a positive significant relationship, where studied parameters were interrelated between them and probably originated from the same sources. Finally, Principal component analysis results showed that anthropogenic activities (agrochemical application, contaminated feeds, and atmospheric particulates from automobile emissions) (Pandey et al., 2012) followed by natural sources (geochemical evaluation) were the main controlling factors for the study area from where the vegetables and fish samples were collected. PCA has shown that the distribution of similar kinds of heavy metals in fish and vegetables was not related which might be due to the variation of emission activities of metals and metalloids from the source to the environment. Additionally, the results of PCA are validated using the CA and PCM analysis, which also match with previous findings of Islam et al. (2016).

### 3.8 SOM analysis result

**Figure 5:**
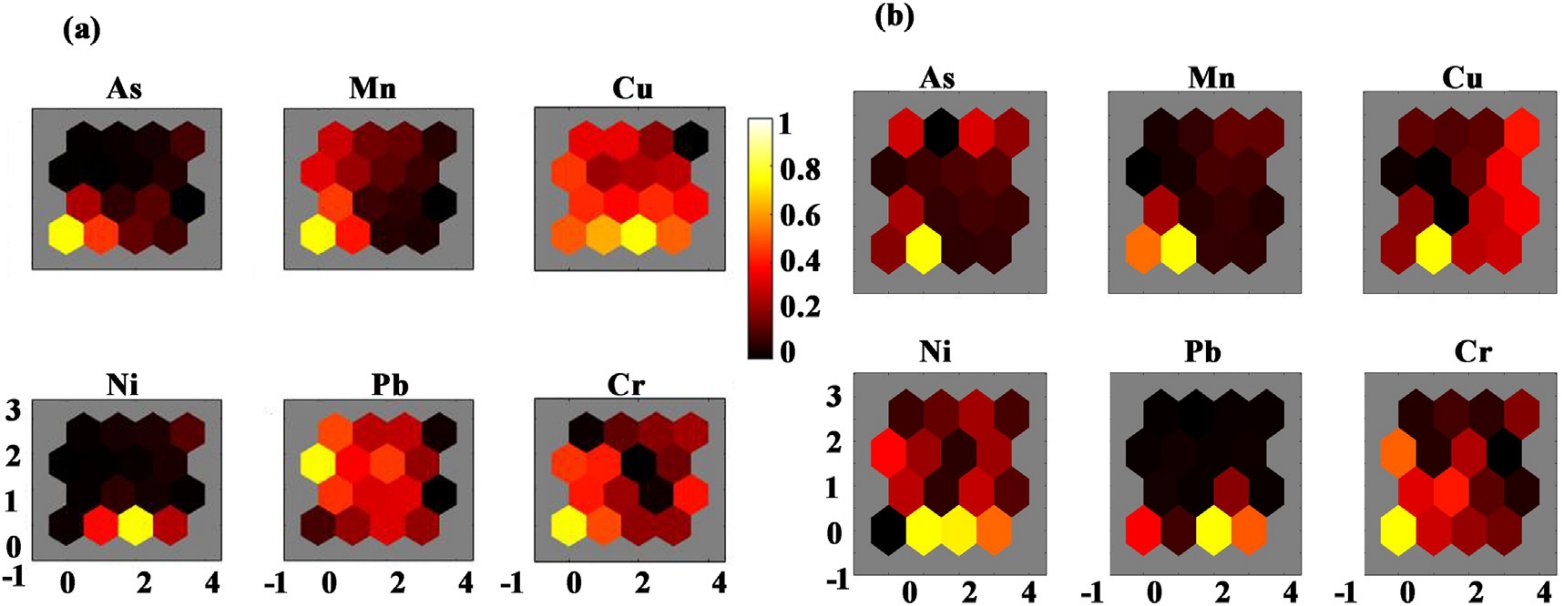
SOM map of concentration of metals and metalloids in (a) vegetable, and (b) fish samples.

The module planes of SOM analysis are presented in Figure 5(a and b), where every variable resembled as given in Figure 5(a and b). The smaller the space of the hexagon is added similar the features of the samples are. In a component plane, an analogous color has shown a positive association between components, while diverse colors stated negative associations. For vegetables three patterns are produced by SOM analysis, the lower left part of the maps displayed higher As, Mn, and Cu, whereas Ni was highly concentrated between the middle part of the left and right side (Figure 5a). Lastly, Pb and Cr were highly concentrated at the lower and middle left part. For the fish dataset, three spatial forms are found. First, As, Mn and Cr exhibited a similar pattern of concentration (highly concentrated in the lower left part). Second, dissimilar to other heavy metal contents, Cu and Ni showed a horizontal gradient (the middle part between the left to the right side) in color shapes, representing that both heavy metals are regulated by diverse processes from those influencing the key components.

Third, Pb values express additional complex color shapes than other components with growing from the lower left to the upper left sides (Figure 5b).

### 3.9 PMF analysis

**Figure 6:**
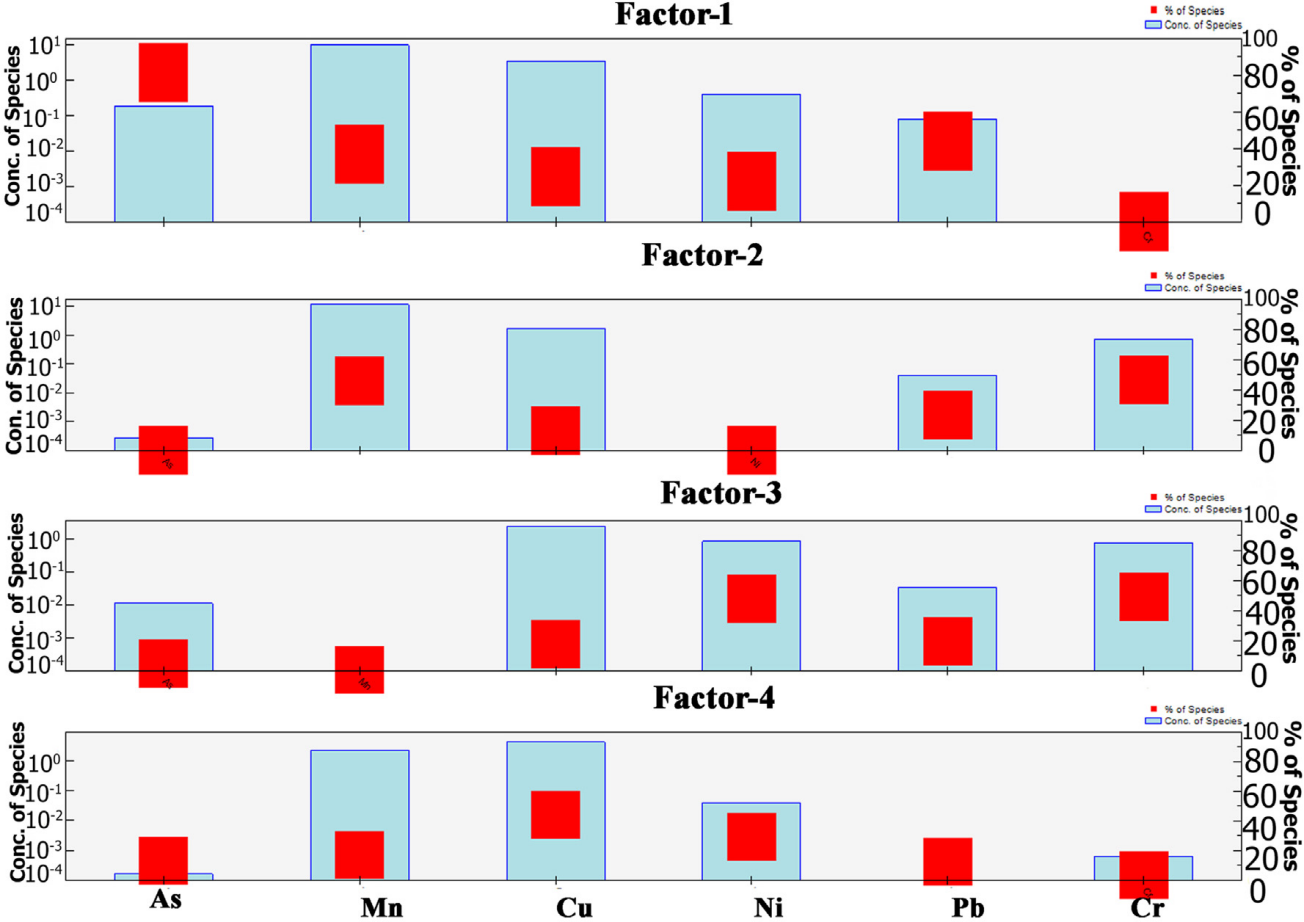
Profiles and contributions of sources of metals and metalloids in vegetable samples from PMF model.

PMF (version 5.0) is a widely used factorization receptor model, to detect and measure the possible source of metals and metalloids in vegetable and fish samples and also to define the contribution of every heavy metal. In this study, for model validation and fitness Q was reduced to regulate the residual matrix and the residuals located between -1 to +1. This system was run 20 times for attaining a good result, where different factors (3 or 5) were tested. The relationship between observed (R^2^) and predicted value varied from 0.67 (Ni) to 0.99 (As) denoted as strong relation and 0.01 (Pb) is defined as lower relation for vegetable samples while the correlation (R^2^) values ranging from 0.51 (Ni) to 0.99 (As) is called strong relation and 0.33 (Cr) shows slightly lower relation for fish samples (Table S2). Consequently, studied heavy metals were well distributed by the PMF model, and the results were reliable. For vegetable species, factor 1 was controlled by As and Pb with loadings of 87.9% and 44.3%, respectively (Figure 6).

The concentration of As and Pb in almost all of the vegetables exceeds the MAC level. Arsenic and Pb in the vegetable species came from the contaminated soil which remarkably notable the natural and mammade sources of As and Pb. Arsenic probably comes from As contaminated groundwater irrigation and excess application of As-rich agrochemicals (Pfeiffer et al., 1991). Lead is generally derived from emissions from fossil fuels, and road dust deposition in agricultural soil (Cheng et al., 2020). Factor 2 was greatly loaded with Cr (46.6%) and Mn (46%). The mean value of Mn and Cr was greater than the recommended safe level, representing that these metals in the soil of vegetable bases come from human activities (Kuerban et al., 2020). Factor 3 was designated by Ni and Cr with the loadings of 48%, and 49.60%, respectively (Figure 6). Ni and Cr were resulting from anthropogenic activities (Zhang et al., 2016), as well as from geochemical weathering (Nicholson et al., 2003). Factor 4 was defined by Cu (44.4%), demonstrating that this metal in the soil had accumulated to vegetable species due to the application of organic and chemical fertilizer and pesticides to continue and increase the soil fertility and assurance the yield (Kuerban et al., 2020; Bhuiyan et al., 2021; Xiong et al., 2010).

**Figure 7:**
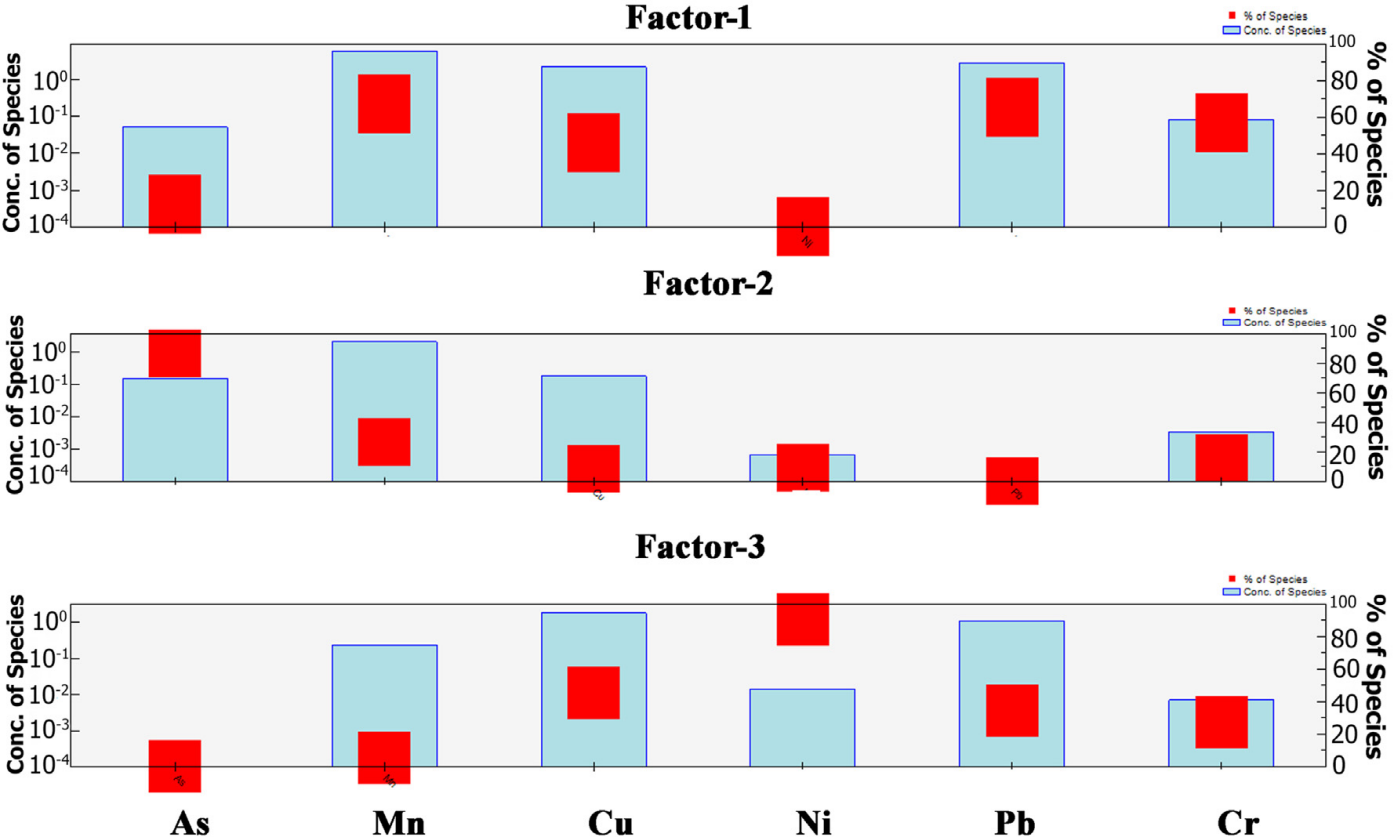
Profiles and contributions of sources of metals and metalloids in fish samples from PMF model.

For fish species, factor 1 was controlled by Mn (67.6%), Pb (65.3%), Cr (57.1%), and Cu (46.2%). A large variation of Mn, Pb, and Cu were the most dominant metal among the metals (Figure 7) might propose a stronger anthropogenic influence (Sarkar et al., 2022). Factor 2 was dominated by As with the loadings of 86.9% (Figure 7), which probably comes from agricultural runoff, and geochemical sources. Factor 3, Ni, had a high factor loading value of 90.50%. The risky contamination of Ni in the fish feed samples was detected by Sarker et al. (2022) where this high content may move in the fish via the raw fish feed.

### 3.10 Limitations of the study

This study shows vital indication about metals and metalloids contamination in fish and vegetable species in the high Ganges river flood plain agro-ecological area in Bangladesh. The samples were collected only in one season and no time-based variability was considered. Additionally, bio-accessibility of metals and metalloids in food items were not detected and thus could not appraisal actual metals and metalloids exposure. Besides, using the risk assessment on these measurements is consequently even an extra compelling. We stated non-carcinogenic and carcinogenic risk of metals and metalloids in terms of a hazard quotient (HQ), and hazard index (HI) and carcinogenic risk (CR), where other food sources were not considered. However, we evaluated six different heavy metals in 15 different vegetables and 11 different fish species. Due to resource constrain this study could not include other vegetable and fish species and heavy metals. Future work should be overwhelmed with this issue.

## 4 Conclusions

This present study assesses the concentrations of six metals and metalloids (As, Mn, Cr, Cu, Ni, and Pb), their sources in commonly consumed fish and vegetable species, and probable human health risks in the Jashore district of Bangladesh. Heavy metal concentrations were significantly varied in vegetable species might be due to the variation of plant growth locations and uptake mechanisms and diverse ingestion behavior in fish species. The concentration of As, Pb, and Cr in vegetable species and Pb and cu in fish species exceeded the maximum allowable concentrations (MAC). According to study findings *Lactuca sativa*, *Cucurbita moschata, Amaranthus gangeticus* and *Channa punctate, Oreochromis mossambicus*, *Dendrobranchiata* are the most contaminated vegetable and fish species, respectively. The multivariate analysis results (PMF, SOM, PCA) recommend that metals and metalloids in fish and vegetable species available in the study area came from different origins and/or under diverse manufacturing systems. The target hazard quotient (THQ) of single metal (except As for vegetables and Pb for fish species) would not pose any probable health risk, but combined metals (HI > 1) pose a momentous risk to the vegetable and fish consumers. The As and Pb associated TCR due to vegetable and fish ingestion showed both untolerable (>1E-04) and tolerable (1E-04 to 1E-06) health risks, respectively. Additionally, probable health risk indicates that 95% of inhabitants in the study area have a substantial chance of As associated cancer risk due to ingestion of vegetable and fish species. Finally, this study recommended that frequent monitoring is necessary for the control and avoidance of metals and metalloids contamination in food items, to ensure food safety and as well as to reduce human health risks allied with heavy metals contamination for the inhabitants of the study area. Additionally, these study outcomes could provide essential recommendations for the farmer on optimum uses of agrochemicals and changing of food consumption pattern for consumers.

## Declarations

### Author contribution statement

Tapos Kumar Chakraborty; Samina Zaman: Conceived and designed the experiments and Wrote the paper.

Gopal Chandra Ghosh: Conceived and designed the experiments and Contributed reagents, materials, analysis tools or data.

Md Ripon Hossain; Md. Shahnul Islam; Ahsan Habib; Md. Simoon Nice: Performed the experiments.

Abu Shamim Khan: Contributed reagents, materials, analysis tools or data.

### Funding statement

This research did not receive any specific grant from funding agencies in the public, commercial, or not-for-profit sectors.

### Data availability statement

Data included in article/supp. material/referenced in article.

### Declaration of interest’s statement

The authors declare no conflict of interest.

### Additional information

No additional information is available for this paper.
