# Supplementary material for: Human health risk ​and receptor model-oriented sources of heavy metal pollution in commonly consume vegetable and fish species of high Ganges river floodplain agro-ecological area, Bangladesh
Source: Heliyon. 2022 Oct 21;8(10):e11172. doi: 10.1016/j.heliyon.2022.e11172 (PMC9618996; doi:10.1016/j.heliyon.2022.e11172)
Supplement: Supplementary material — . [file mmc1.docx]

**Supplementary material**

**Human health risk assessment and receptor model-oriented sources of heavy metal pollution in commonly consume vegetable and fish species of high Ganges river floodplain agro-ecological area, Bangladesh**

Tapos Kumar Chakraborty^a^*, Gopal Chandra Ghosh^a^, Md Ripon Hossain^a^, Md. Shahnul Islam^a^, Ahsan Habib^a^, Samina Zaman^a^, Himel Bosu^a^, Md. Simoon Nice^a^, Monisankar Haldar^b^, Abu Shamim Khan^c^

^a^Department of Environmental Science and Technology, Jashore University of Science and Technology, Jashore 7408, Bangladesh

^b^Department of Computer Science and Engineering, Jashore University of Science and Technology, Jashore 7408, Bangladesh

^c^Environmental Laboratory, Asia Arsenic Network, Arsenic Center, Benapole Road, Krishnobati, Pulerhat, Jashore 7400, Bangladesh

Table S1. Concentrations of metals found in certified reference materials DORM-4 from National Research Council Canada (means ± standard errors, in mg/kg as wet wt.) by AAS.

| Elements | Certified value | Observed value | Recovery (%) |
| --- | --- | --- | --- |
| As | 6.80±0.64 | 6.29±0.07 | 92 |
| Ni | 1.36±0.22 | 1.50±0.09 | 110 |
| Mn | 11.2 ±0.4 | 9.85±0.160 | 88 |
| Cr | 1.87±0.16 | 1.70±0.10 | 90 |
| Cu | 15.9±0.9 | 14.96±0.80 | 93 |
| Pb | 0.416±0.053 | 0.386±0.09 | 92 |

Table S2. PMF model accuracy data

| Species | Elements | Category | R^2^ | Intercept | Intercept SE | Slope | Slope SE | SE | Normal Resided |
| --- | --- | --- | --- | --- | --- | --- | --- | --- | --- |
| Fish | As | Strong | 0.999 | -0.009 | 0.004 | 1.047 | 0.005 | 0.011 | Yes |
|  | Mn | Strong | 0.999 | 0.433 | 0.114 | 0.933 | 0.010 | 0.224 | Yes |
|  | Cu | Strong | 0.986 | 0.312 | 0.183 | 0.922 | 0.036 | 0.219 | Yes |
|  | Ni | Strong | 0.510 | 0.040 | 0.020 | 0.368 | 0.120 | 0.059 | Yes |
|  | Pb | Strong | 0.669 | -2.015 | 1.500 | 1.392 | 0.326 | 0.862 | Yes |
|  | Cr | Strong | 0.326 | 0.045 | 0.048 | 0.623 | 0.298 | 0.048 | Yes |
|  |  |  |  |  |  |  |  |  |  |
| Vegetable | As | Strong | 0.992 | -0.014 | 0.006 | 1.074 | 0.026 | 0.014 | Yes |
|  | Mn | Strong | 0.979 | 1.906 | 1.124 | 0.910 | 0.036 | 2.335 | Yes |
|  | Cu | Strong | 0.959 | -0.781 | 0.856 | 1.056 | 0.060 | 0.926 | Yes |
|  | Ni | Strong | 0.674 | 0.978 | 0.171 | 0.402 | 0.077 | 0.295 | Yes |
|  | Pb | Strong | 0.010 | 0.172 | 0.023 | 0.005 | 0.013 | 0.082 | Yes |
|  | Cr | Strong | 0.982 | -0.036 | 0.064 | 1.021 | 0.038 | 0.098 | Yes |

Table S3. Correlation coefficient matrix for metals and metalloids in vegetable and fish species collected from Jashore district, Bangladesh.

| Species |  | As | Mn | Cu | Ni | Pb | Cr |
| --- | --- | --- | --- | --- | --- | --- | --- |
| Vegetable | As | 1 |  |  |  |  |  |
|  | Mn | 0.684** | 1 |  |  |  |  |
|  | Cu | 0.744** | 0.598* | 1 |  |  |  |
|  | Ni | 0.391 | 0.182 | 0.441 | 1 |  |  |
|  | Pb | -0.228 | 0.255 | -0.066 | 0.316 | 1 |  |
|  | Cr | -0.104 | 0.518* | -0.197 | -0.157 | 0.538* | 1 |
|  |  |  |  |  |  |  |  |
| Fish | As | 1 |  |  |  |  |  |
|  | Mn | 0.767** | 1 |  |  |  |  |
|  | Cu | 0.315 | .188 | 1 |  |  |  |
|  | Ni | 0.066 | -0.270 | 0.594* | 1 |  |  |
|  | Pb | -0.371 | 0.114 | 0.231 | -0.068 | 1 |  |
|  | Cr | 0.616* | 0.650* | 0.156 | -0.101 | -0.228 | 1 |

**. Correlation is significant at the 0.01 level (2-tailed).

*. Correlation is significant at the 0.05 level (2-tailed).


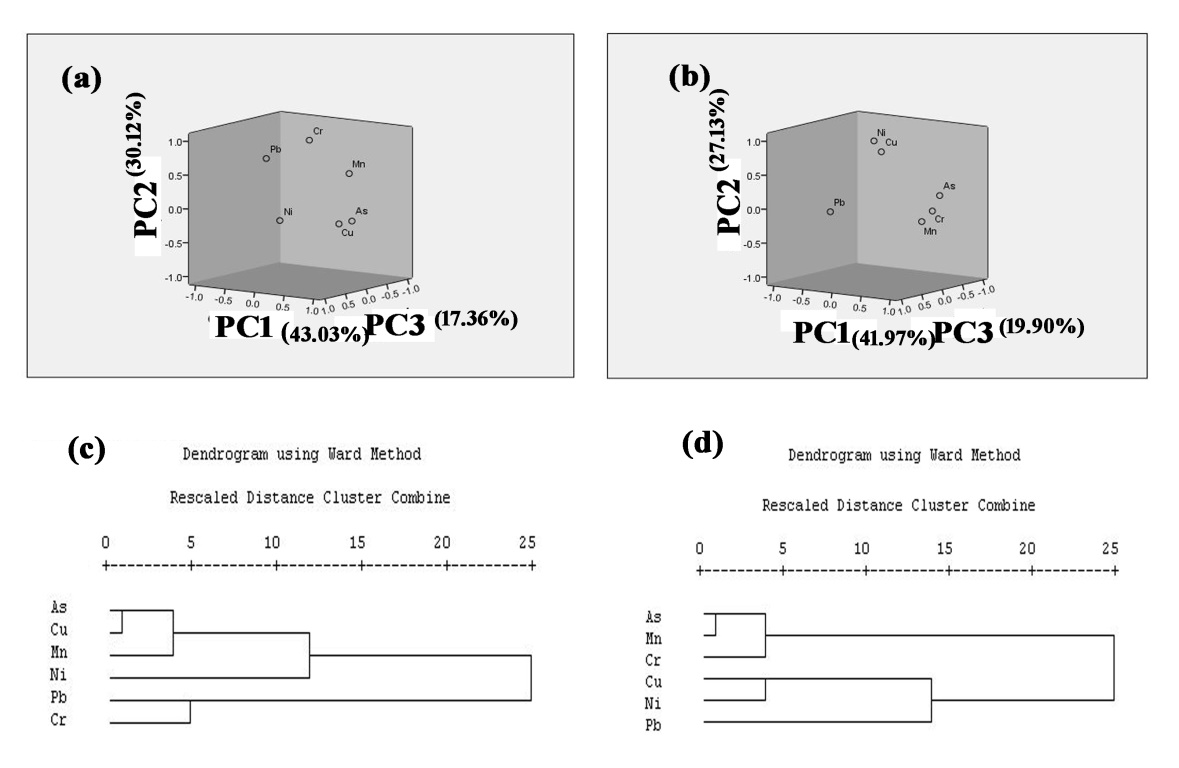


**Figure S1**. PCA (a) Vegetables, (b) fish and cluster diagram (c) Vegetables, (d) fish
